# Leave of absence due to depression*

**DOI:** 10.1590/1518-8345.3634.3274

**Published:** 2020-06-01

**Authors:** Carla Danielle Araújo Feitosa, Márcia Astrês Fernandes

**Affiliations:** 1 Universidade Federal do Piauí, Colégio Técnico de Bom Jesus, Bom Jesus, PI, Brazil.; 2 Universidade Federal do Piauí, Departamento de Enfermagem, Teresina, PI, Brazil.

**Keywords:** Occupational Health, Mental Health, Social Security, Sick Leave, Absenteeism, Depression, Saúde do Trabalhador, Saúde Mental, Previdência Social, Licença Médica, Absenteísmo, Depressão, Salud Laboral, Salud Mental, Seguridad Social, Ausencia por Enfermedad, Absentismo, Depresión

## Abstract

**Objective:**

to analyze the occurrence of leave of absence due to depression among workers.

**Method:**

census, descriptive-analytical study, with retrospective collection. Population composed of 2,267 workers on leave due to depression with data from the Unified Benefits Information System. The independent variables were: sex, age group, income; county, origin, number of leaves of absence and type of benefit. For data analysis, descriptive statistics were used and the chi-square and Fisher’s exact tests were applied. The variables that presented a value of p≤0.20 were submitted to logistic regression.

**Results:**

there was a predominance of females, age ≥50 years, from the capital, with income of one to two minimum wages, urban origin and single removal. Single leaves of absence occurred mainly due to a mild depressive episode and the benefit granted to the significant majority was social security sickness benefit. Among those who were on leave of absence more than once, the main cause was recurrent depressive disorder, a current mild episode and, in terms of benefit, social security sickness benefit. There was a statistical association between total time and absence from work. In logistic regression, it was found that the time ≥60 days, was 3.1 times longer in recurrent depressive disorder.

**Conclusion:**

there were an expressive quantitative number of absences due to depression, in which it was observed, especially, that the absence time remained long.

## Abstract (pt)

**Objetivo:**

analisar a ocorrência de afastamentos das atividades laborais motivados pela depressão.

**Método:**

estudo censitário, descritivo-analítico, com coleta retrospectiva. População composta por 2267 trabalhadores afastados por depressão com dados procedentes do Sistema Único de Informações de Benefícios. As variáveis independentes foram: sexo, faixa etária, renda, município, procedência, quantidade de afastamentos e tipo de benefício. Para a análise dos dados, utilizou-se a estatística descritiva e foram aplicados os testes qui-quadrado e exato de Fisher. As variáveis que apresentaram valor de p≤0,20 foram submetidas à regressão logística.

**Resultados:**

houve a predominância do sexo feminino, idade ≥50 anos, da capital, com renda de um a dois salários mínimos, procedência urbana e único afastamento. Os afastamentos únicos ocorreram, principalmente, por episódio depressivo leve e o benefício concedido para a expressiva maioria foi o auxílio-doença previdenciário. Entre os que se afastaram mais de uma vez, a principal causa foi o transtorno depressivo recorrente, episódio atual leve e, quanto ao benefício, foi o auxílio-doença previdenciário. Houve associação estatística entre o tempo total e os afastamentos ao trabalho. Na regressão logística, verificou-se que o tempo ≥60 dias foi 3,1 vezes maior no transtorno depressivo recorrente.

**Conclusão:**

verificou-se um quantitativo expressivo de ocorrência de afastamentos devido à depressão no qual se observou, especialmente, que o tempo de afastamento manteve-se longo.

## Abstract (es)

**Objetivo:**

analizar la ocurrencia de ausencias laborales por depresión.

**Método:**

estudio censal, descriptivo analítico, con recogida de datos retrospectiva. Población compuesta por 2267 trabajadores con ausencias laborales por depresión, con datos procedentes del Sistema Único de Informaciones de Beneficios. Las variables independientes fueron: sexo, intervalo etario, renta, municipio, procedencia, cantidad de licencias médicas y tipo de beneficio. Para el análisis de los datos, se utilizaron estadística descriptiva y se aplicaron las pruebas chi-cuadrado y exacta de Fisher. Las variables que presentaron valor de p≤0,20 fueron sometidas a regresión logística.

**Resultados:**

predominó el sexo femenino, edad ≥50 años, de la capital, renta de uno a dos salarios mínimos, procedencia urbana y única ausencia laboral.. Principalmente, las ausencias únicas ocurrieron por episodio depresivo leve y el beneficio, para la expresiva mayoría, fue el auxilio por enfermedad de la seguridad social. Entre los que obtuvieron licencias médicas más de una vez, la principal causa fue el trastorno depresivo recurrente, episodio actual leve; en cuanto al beneficio, el auxilio por enfermedad fue de la seguridad social. Hubo asociación estadística entre el tiempo total y las ausencias laborales.. En la regresión logística, se verificó que el tiempo ≥60 días fue 3,1 veces mayor en el trastorno depresivo recurrente.

**Conclusión:**

se verificó una cantidad expresiva de ocurrencia de ausencias, debido a la depresión, en la cual se observó, especialmente, que el tiempo de ausencia se mantuvo extenso.

## Introduction

Work has been present in society since the dawn of humanity, presenting different meanings according to the context and the period in which it is described. The expansion of the capitalist model is characterized by its presentation as a profitable source, in which there was an expressive increase in the time and dedication spent by the worker, so that his craft started to occupy a central place in their life^(^ 1 ^)^.

It can be seen that the pattern of work organization has been modified; generating new ways of producing social vulnerabilities in which issues related to health and work have emerged. The causal relationship between these factors broadened the view of the need for protective measures for workers’ health^(^ 2 ^)^.

Work-related illnesses, derived from emotional stress, arise when the individual is exposed to risks generated by the activities he or she develops, so that the mental and behavioral disorders resulting from work can be triggered by several reasons, ranging from the pace of work excessive, aiming at productivity, even the labor relations based on authoritarianism. The work can act as a provoker of a pre-existing psychological disease or of a still latent disorder, presenting a multi-causal etiology in which sets of diverse factors interact in a complex way^(^ 3 ^-^ 4 ^)^.

In Brazil, mental illness among workers has been configured as the third cause of absence from work, being equivalent to sickness benefits due to work incapacity^(^ 5 ^)^. Among the mental disorders that affect this population, there are depressive episodes.

The estimated total number of people living with depression has increased by 18.4% between 2005 and 2015. Brazil has 11,548,577 people with depression, second only to the United States on the American continent^(^ 6 ^)^. And, when it comes to the relationship with work activities, the World Health Organization (WHO) predicts that, in 2020, the disorder will occupy the second cause of leave of absence in the world^(^ 7 ^)^.

In the context of the state of Piauí, when evaluating the year 2014, it was observed that depression, among mental and behavioral disorders, is the fourth cause of the highest average number of days off work^(^ 8 ^)^.

In this perspective, it is emphasized that this disease causes, in the worker, losses, such as disability, reduced productivity and removal from employment. It has also been shown that the costs of loss of productivity associated with depression far exceed the costs of resources used to treat and control the disorder; therefore, the observation and prevention of this disease are of fundamental importance^(^ 1 ^-^ 9 ^)^.

The study also corroborates the awakening in the attention to Nursing professionals, especially in the field of occupational health and mental health, in a way that highlights the need for research and intervention in the workplace with regard to their organization, as highlight some authors^(^ 10 ^-^ 11 ^)^, in order to contribute to its role in promoting mental health.

In general, the affliction by depression has taken worldwide proportions, including among workers, with the disease being pointed out as a source of suffering, stigma and exclusion, interfering adversely in work performance. In this perspective, a profound reflection on the occurrence of leave of absence due to depression among workers is increasingly necessary in order to add new perspectives and expand knowledge on the subject. Thus, the objective of this study is to analyze the occurrence of leave of absence due to depression among workers.

## Method

This is a census, descriptive-analytical study, with retrospective collection. The research was carried out at the headquarters of the National Social Security Institute (INSS abbreviation in Portuguese), located in the city of Teresina, state of Piauí, Brazil, where data were collected from workers of that state, on leave of absence due to depression, registered in the Unified Benefit Information System (SUIBE - abbreviation in Portuguese), between 2010 and 2015.

It should be noted that this time frame was chosen with a view to the perspectives of the World Health Organization, that depression would be the second cause of lost work days due to illness in the world^(^ 7 ^)^, as well as the study started at the beginning of 2016, when the database on the health of the worker was still open, thus determining the year 2015 as the final period for use in analyses, with use therefore, of the most organized and recent data.

The collection took place from March to May 2017, being carried out using a form adapted by the researchers responsible for the study according to the only variables contemplated by the INSS database. These variables consisted of sociodemographic and occupational data - such as sex, age in complete years, income (categorized by the database), municipality and origin (urban or rural) - and data related to leave of absence - “there was more than one leave of absence” (yes or no), number of leaves of absences, length of leave (days), type of benefit (social security sickness benefit, accidental sickness benefit, retirement due to accidental disability and retirement due to social security disability) and the cause of the leave of absence according to the type of depression according to the International Disease Code (ICD-10).

As inclusion criteria, we adopted data on workers on leave of absence and disability pensions who presented illnesses in code F of the ICD-10 and that varied from F 32.0 to F 33.9, in the period from 2010 to 2015. As exclusion criteria, the leave of absence of workers with incomplete data on the variables analyzed, were chosen.

The inference that depression leave of absence is directly related to work or not can be performed according to the type of benefit indicated.

Initially, the INSS statistical data were requested to the IT sector manager and a spreadsheet was obtained, in Microsoft Excel format, a report with the quantitative and available variables referring to leave of absence and disability retirement due to depression, in the time frame from 2010 to 2015.

Soon after, in order to obtain the data related to the number of leave of absences per worker, the filtering by date of birth was carried out in which the repetition of information of some of them was observed. After selecting the database, each of the 2,267 workers was analyzed with information contained in SUIBE.

It is noteworthy that, when observing the time off for workers who retired during the investigated time frame, it was found that SUIBE considered it to be zero. However, given the importance of this information and the high number of retired workers due to depression, it was decided to maintain, for the analysis, the two types of disability pensions. Such measure is also justified by the fact that the disability retirement, unlike the others, is not considered lifelong since the retiree has canceled this benefit if he, voluntarily, returns to the activity^(^ 12 ^)^.

The data was entered into the Excel 2010 software and later exported to the Statistical Package for the Social Science software, version 20.0. The double entry technique was used to check and clean the database. Soon after, descriptive analyzes (frequencies, measures of central tendency and dispersion) of the research variables were performed.

For the bivariate analysis, the dependent variable was categorized as <60 days and ≥60 days so that this time off was adopted because it uses a reference that emphasizes that two months of leave of absence, in most cases, can correspond to insufficient time for the worker’s full recovery^(^ 13 ^)^. In addition, the age group variable (<40 years; ≥40 years) was carried out.

For the calculation of the occurrence of depression and for bivariate analyzes, considering workers who left more than once, it was decided to use the last cause of leave of absence, as well as the type of corresponding benefit.

For that, based on the above, we opted for the use of Pearson’s chi-square test and, when its assumptions were violated, Fisher’s exact test was used. The variables that, in the bivariate analysis, had a value of p ≤ 0.20 were submitted to the multivariate model of logistic regression, here called adjusted odds ratio.

For the other analyses, the significance level of p ≤ 0.05 was maintained and the confidence interval was set at 95%.

The research was approved by the Executive Management of the INSS in the state of Piauí and approved by the Research Ethics Committee of the Federal University of Piauí through opinion number 1,827,564. In addition, it obeyed all ethical precepts set forth in Resolution No. 466/12 of the National Health Council (NHC).

## Results

According to the characterization presented in Table 1, of the 2267 workers on leave of absence, 63.6% were female, with a mean age of 47.2 (± 11.1), of which 44.2% belonged to the age group of 50 years or more. As for the municipality of residence, 47.9% were from Teresina, capital of Piauí. Of the total, it was observed that 77.1% of workers had income ranging from one to two minimum wages. Regarding the origin, it was found that 80.9% were of urban origin. With regard to the number of leaves of absence, 80.9% left only once and 19.1% had more than one leave of absence. Of these, 16.1% left twice, 0.1% had five leaves of absence and 19.1% of them left more than once.

**Table 1:** Sociodemographic, economic and quantitative characterization of leaves of absence due to depression among workers. Teresina, PI, Brazil, 2010-2015 (n=2267)

Variables	N	%
Sex		
Male	825	36.4
Female	1442	63.6
Age group (years)		
20 to 29	111	4.9
30 to 39	533	23.5
40 to 49	622	27.4
50 or more	1001	44.2
Municipality		
Teresina	1085	47.9
Other municipalities in the interior of Piauí	1031	45.5
Other states	151	6.7
Income (minimum wage)*		
1 to 2	1747	77.1
2 to 3	234	10.3
3 to 4	108	4.8
4 to 5	72	3.2
5 to 6	42	1.9
More than 6	64	2.8
Origin		
Urban	1834	80.9
Rural	433	19.1
There was more than one leave of absence		
Yes	434	19.1
No	1833	80.9
Number of leaves of absence		
One	1833	80.9
Two	364	16.1
Three	53	2.3
Four	14	0.6
Five	3	0.1

Source – SUIBE.

* Minimum wage in Brazil, 2015 = R$788.00 reais per month or US$ 203.62 US dollars monthly

Among workers who had only one leave of absence, it was found that, in 94.3% of cases, the type of benefit granted was social security sickness benefit. As for the cause of absence according to the type of depression, 24.5% were diagnosed with a mild depressive episode followed by a moderate depressive episode (16.5%) and a recurrent depressive disorder and a current mild episode (15.5%). Regarding the time off, 29.9% of workers were absent for more than 120 days, with an average of 100.9 days (± 56.5).

Regarding the types of benefits granted to workers with more than one leave of absence, it was found that, in the first (95.2%), in the second (68.2%), in the third (62.9%), in the fourth (52.9%) and in the fifth leave of absence (66.7%), the type of benefit most granted was social security sickness benefit. Among these workers, 28.8% retired due to social security disability in the second leave of absence, 37.1% in the third and 41.2% in the fourth.

As to the cause of the leave of absence according to the type of depression for workers who had left more than once, it was identified that, in the first (21.9%), in the second (27.6%), in the third (32.4 %) and in the fourth (35.3%) leave of absence, most workers presented recurrent depressive disorder, a current mild episode. As for the other types of depression, recurrent depressive disorder, a current serious episode with psychotic symptoms, was highlighted in the second (14.3%), in the third (19.7%), in the fourth (23.5%) and in fifth leave of absence (33.3%).

When describing the time off in days of workers with more than one occurrence, it was found that, in the first leave of absence, 34.6% left for more than 120 days, with an average of 102.9 days (± 52.1). As for the second and third leaves of absence, 33.2% and 37.1%, respectively, were absent from work for up to 30 days, with an average of 80.1 days (± 77.0) for the second and 85.4 days (± 100.7) for the third. Regarding the time of the fourth leave of absence, 41.2% left for up to 30 days and 41.2% for a period above 120 days, with an average of 93.1 days (± 99.0).

In Table 2, when the crossing between the total time away from work and the sociodemographic variables of the study was performed, a statistically significant association was observed between some variables. In the age group (p <0.001), it was found that individuals aged 40 years or more tend to stay away from work for longer. Regarding income (p <0.017), those who earn up to two minimum wages have a greater tendency to leave of absence for a longer time and, regarding their origin (p <0.01), workers from the rural area stay away for a longer time.

**Table 2:** Association of the total time away from workers with sociodemographic characteristics. Teresina, PI, Brazil, 2010-2015 (n=2267)

Variables	<60 days		≥60 days		p-value*
	N	%	N	%	
Sex					
Male	149	18.1	676	81.9	0.124
Female	299	20.7	1143	79.3	
Age group (years)					
<40	208	28.7	516	71.3	<0.001
≥40	240	15.6	1303	84.4	
Income (minimum wage)^†^					
1 to 2	318	18.2	1429	81.8	0.017
2 to 3	62	26.5	172	73.5	
3 to 4	23	21.3	85	78.7	
4 to 5	16	22.2	56	77.8	
5 to 6	12	28.6	30	71.4	
More than 6	17	26.6	47	73.4	
Origin					
Urban	400	21.8	1434	78.2	<0.01
Rural	48	11.1	385	88.9	

Source - SUIBE;

* Pearson's chi-square test;

† Categorized by the Single Benefit Information System: minimum wage in Brazil, 2015 = R$788.00 reais *per* month or US$ 203.62 US dollars monthly

In the association of the total time off with the types of depression (Table 3), there was a statistically significant association. For the mild depressive episode (p <0.001), it was observed that the workers spent less time away from those who did not present this condition. Regarding severe depressive episodes with psychotic symptoms (p = 0.005), recurrent depressive disorder, mild current episode (p <0.001) and recurrent depressive disorder, severe current episode with psychotic symptoms (p <0.001), it was found that workers were away longer than those who did not present these diagnoses.

**Table 3:** Association of total time off work for workers with types of depression. Teresina, PI, Brazil, 2010-2015 (n=2267)

Variable	<60 days	≥60 days	p-value
	N(%)	N(%)	
Mild depressive episode			
Yes	173(35.5)	315(64.5)	<0.001*
No	275(15.5)	1504(84.5)	
Moderate depressive episode			
Yes	75(21.9)	268(78.1)	0.161*
No	373(19.4)	1551(80.6)	
Severe depressive episode no psychotic symptoms			
Yes	30(13.0)	201(87.0)	0.006*
No	418(20.5)	1618(79.5)	
Severe depressive episode with psychotic symptoms			
Yes	16(10.9)	131(89.1)	0.005*
No	432(20.4)	1688(79.6)	
Other depressive episodes			
Yes	2(50.0)	2(50.0)	0.128^†^
No	446(19.7)	1817(80.3)	
Depressive episode not specified			
Yes	6(17.1)	29(82.9)	0.695*
No	442(19.8)	1790(80.2)	
Recurrent depressive disorder, current mild episode			
Yes	52(12.8)	354(87.2)	<0.001*
No	396(21.3)	1465(78.7)	
Recurrent depressive disorder, current moderate episode			
Yes	42(23.1)	140(76.9)	0.242*
No	406(19.5)	1679(80.5)	
Recurrent depressive disorder, serious current episode without psychotic symptoms			
Yes	31(15.3)	171(84.7)	0.099*
No	417(20.2)	1648(79.8)	
Recurrent depressive disorder, severe current episode with symptoms psychotic			
Yes	14(7.8)	165(92.2)	<0.001*
No	434(20.8)	1654(79.2)	
Recurrent depressive disorder currently in remission			
Yes	5(38.5)	8(61.5)	0.089*
No	443(19.7)	1811(80.3)	
Other depressive disorders Recurrent			
Yes	0(0.0)	17(100.0)	0.999^†^
No	448(19.9)	1802(80.1)	
Depressive disorder unspecified recurrent			
Yes	2(10.0)	18(90.0)	0.271^†^
No	446(19.8)	1801(80.2)	

Source - SUIBE;

* Pearson's chi-square test;

† Fisher's exact test

At the intersection of the types of benefits granted to workers with the total time off (Table 4), a statistically significant association was identified with the accident-related sick pay (p = 0.001). These workers were less time away compared to those who do not have this type of assistance. For retirement due to social security disability (p = 0.002), it was found that those who have this benefit tend to stay longer.

**Table 4:** Association of the total time away from workers with the types of benefits granted. Teresina, PI, Brazil, 2010-2015 (n=2267)

Variable	<60 days		≥60 days		p-value
	N	%	N	%	
Sickness benefit social security					
Yes	395	19.8	1597	80.2	0.828*
No	53	19.3	222	80.7	
Sickness benefit Accident					
Yes	37	32.2	78	67.8	0.001*
No	411	19.1	1741	80.9	
Retirement by accidental disability					
Yes	0	0.0	2	100	0.999^†^
No	448	19.8	1817	80,2	
Retirement due to disability social security					
Yes	16	10.1	142	89.9	0.002*
No	432	20.5	1677	79.5	

Source - SUIBE;

* Pearson's chi-square test;

† Fisher's exact test

In the multiple logistic regression model, it was found that the chance of the absence time being ≥60 days is 0.5 times lower among those individuals who are <40 years old when compared to workers aged ≥40 years. Urban customers are 0.4 times less likely to be away for more than 60 days compared to rural customers (Table 5).

**Table 5:** Logistic regression of long-term leave of absence (≥60 days) with sociodemographic characteristics, caused by type of depression and benefits granted. Teresina, PI, Brazil, 2010-2015

Variables	≥60 days		
	OR* (adjusted)	p-value	CI95%^†^
Age group (years)			
<40	0.5	<0.001	0.4 – 0.6
≥40	1		
Income (minimum wage)			
1 to 2	1.6	0.094	0.9 – 2.9
2 to 3	1.0	0.991	0.6 – 1.9
3 to 4	1.3	0.430	0.6 – 2.7
4 to 5	1.3	0.556	0.6 – 2.8
5 to 6	0.9	0.821	0.4 – 2.2
More than 6	1		
Origin			
Urban	0.4	<0.001	0.3 – 0.6
Rural	1		
Mild depressive episode			
Yes^‡^	0.3	<0.001	2.4 – 3.8
No	1		
Severe depressive episode without psychotic symptoms			
Yes^‡^	1.7	0.007	1.2 – 2.5
No	1		
Severe depressive episode with psychotic symptoms			
Yes^‡^	2.1	0.006	1.2 – 3.5
No	1		
Recurrent depressive disorder, current mild episode			
Yes^‡^	1.8	<0.001	1.3 – 2.5
No	1		
Recurrent depressive disorder, current severe episode with psychotic symptoms			
Yes^‡^	3.1	<0.001	1.7 – 5.3
No	1		
Accidental sickness benefit			
Yes^‡^	0.49	0.001	0.3 – 0.7
No	1		
Retirement due to social security disability			
Yes^‡^	2.3	0.002	1.3 – 3.8
No	1		

Source - SUIBE;

* OR = adjusted odds ratio;

† 95% CI = 95% Confidence Interval;

‡ Reference category. The p value was obtained by logistic regression

In workers who presented recurrent depressive disorder, a current severe episode with psychotic symptoms, the chance of being away for ≥60 days was 3.1 times greater than those who did not have this cause, whereas, for participants with recurrent depressive disorder, a current mild episode, there was a 1.8-fold greater chance of leaving for ≥60 days compared to those who did not have this diagnosis (Table 5).

The total time away from work for workers who were absent for a period of ≥60 days was 2.3 times greater among those who, at the end, presented retirement due to social security disability (Table 5).

## Discussion

Of the workers on leave of absence in the state of Piauí, the majority were female. A similar result was found in a longitudinal study of mental health and labor relations conducted with 4,427 Swedish workers^(^ 14 ^)^. Another investigation carried out in Sweden, with workers diagnosed with depression or anxiety disorder, revealed that women made up 70.4% of the sample^(^ 15 ^)^.

The average age corresponded to 47.2 years, of which the majority belonged to the age group aged 50 or over. This data is similar to that obtained in research carried out in South Korea, in which the working population with depressive symptoms had an average age of 47.85 years^(^ 16 ^)^. These data reflects the current demographic transition, in which the aging population is seen, as well as conjecture the current social security and economic situation of the country in which the worker, even if ill, ends up postponing his permanent removal and remains in his job for a longer period of time.

As for the municipality of origin, 47.9% of the workers came from the capital. In a survey carried out in the state of Alagoas, in 2009, 74.1% of medical licenses granted due to leave of absence due to Mental and Behavioral Disorders (MBD) were also due to workers from the state capital^(^ 17 ^)^.

In the screen survey, it was found that 77.1% of workers had an income ranging from one to two minimum wages. This information supports the idea that low income levels may be factors underlying the development of mental disorders, as they impose restrictions on everyday life, reducing the population’s access to essential elements of good mental health.

Regarding the origin, this research identified that 80.9% were urban workers. Similarly, research carried out in Xanxerê, state of Santa Catarina, Brazil, with a view to raising the profile of the insured, also identified a greater number of workers in the urban area in which 68.8% had such a characteristic^(^ 18 ^)^.

When analyzing these findings, it is assumed that the aforementioned class with the highest frequency of absences is more susceptible to the stressors of life in large urban centers, as well as to greater competitiveness and the demands of the labor market.

With regard to the variable “more than one leave of absence”, it is highlighted that 19.1% of the insured persons in Piauí had recurrence. A study carried out on leave of absence for federal civil servants in Palmas, state of Tocantins, Brazil, found that 30.1% of the population studied also had more than one leave of absence due to MBD, of which a significant number was observed due to depressive episodes, reactions severe stress and adaptation disorders^(^ 19 ^)^.

With regard to the number of leaves of absence, it was found that the expressive majority of workers left only once, a fact corroborated by a survey carried out in Japan where the number of single leaves of absence due to MBD was constituted as a majority among workers, so 62.6% had a leave of absence, 23.6% had two, 7.2% had three, 3.0% had four, 2.6% had five and 0.5% six leave of absences^(^ 20 ^)^.

Studies on the subject are still incipient, especially with the use of variables from the INSS, which makes it relevant to share, in the scientific community, such information, considering the increasing concession and maintenance of benefits resulting from psychic disorders.

Among the benefits granted, at the level of Brazil, are pensions, accident allowances, prison allowances, pensions, social protection and sickness benefits. The latter corresponds to the benefit that the Social Security insured receives, temporarily, when he is incapacitated for work, which is subdivided into social security, whose nature is not directly related to work, and in accident, when the leave of absence is related to the activity professional^(^ 21 ^)^.

For those workers who had only one leave of absence, in 94.3% of cases, the type of benefit granted was social security sickness benefit, as well as for those who left more than once. It was found that, in the first, second, third, fourth and fifth leaves of absence, the type of benefit most granted was the social security. In line with these data, it was observed that, in 2015, Social Security granted 4.3 million benefits, of which 88.5% were, also social security, corresponding to the majority of cases^(^ 12 ^)^.

In Piauí, in 2016, when assessing leave of absence due to anxiety disorder, it was also found that social security sickness benefit was the type of benefit most granted (76.7%) to workers^(^ 21 ^)^.

It is also noteworthy that in this state, among workers on more than one leave of absence, 125 (28.8%) retired due to social security disability in the second leave of absence, 26 (37.1%) in the third and seven (41.2%) in the room.

And, although the cases of disability retirement were not assessed for each of the absences in the survey, it is worth mentioning a study carried out in France, identifying that, after previous leave of absence, 529 workers retired. For the authors, the findings suggest that absence due to illness should be considered as a marker risk for future retirement, especially with regard to psychiatric illnesses^(^ 22 ^)^.

In agreement with the findings of this research, a study carried out with 1,013 workers in Italy Italy found that, among them, 17% of the sample had depression. And that, for all the compared categories, the largest proportions regarding the typology were mild depressive episode followed by moderate depressive episode and severe depressive episode^(^ 23 ^)^.

It should be noted that nursing professionals also have high rates of illness, including mental illness. The growing number of dismissals of workers in this category in health institutions, caused by MBD, has caused concern among employers. In this perspective, in a study carried out in Piauí, with 597 nursing professionals, it was found that the moderate depressive episode was the most prevalent among workers on leave^(^ 11 ^)^.

Regarding the length of leave, an investigation carried out in Spain, with workers on leave due to MBD, found that individuals had an average of 147.7 days leave of absence. Those diagnosed with depression had a longer leave^(^ 24 ^)^. Still in Spain, a survey of data on workers who had one or more episodes of temporary leave demonstrated that MBDs had an average absence of 117 days. For depressive disorder, the average duration was 167.9 days^(^ 25 ^)^.

There was a variation in the average of days between the studies analyzed and this research. However, it should be noted that the time off was considered long, thus demonstrating the severity of depressive symptoms and the need for considerably significant time for the improvement and recovery of the worker.

There are several factors that can contribute to the appearance of MBD among workers, especially depression, considering that work-related illnesses, for the most part, do not result from direct trauma, but from a set of causalities and daily psychological constraints, not always noticeable, but that install themselves over time^(^ 13 ^)^. In this perspective, a statistically significant association was observed between time and age, income and origin. The association of these variables was also found in a study developed with workers on leave due to anxiety in the state of Piauí^(^ 21 ^)^.

Cross-sectional research, carried out with data on workers on leave of absence from MBD in state of Santa Catarina, Brazil, among which mood disorders were more prevalent (57.4%), showed a statistically significant correlation between age and time in days on leave of absence for Health treatment^(^ 26 ^)^.

The multivariate analysis showed that the chance of long-term leave was 0.5 (95% CI 0.4 - 0.6) times lower among individuals aged <40 years compared to workers aged ≥40 years.

When investigating absenteeism due to illness for longer periods and relating it to age, it has been shown in the literature that, for older workers, this fact is due to the greater deterioration of health status. In a survey conducted with information from a French database, it was found that age plays an important role in sick leave, concluding that the increase in this variable increases the length of leave^(^ 27 ^)^.

In Finnish public sector workers, a significant difference was found between the time of incapacity for work due to depression and income. Although Finland is a European country with a high socioeconomic status, the income factor has been considered a strong predictor of depressive disorders and even anxiety disorders among workers^(^ 28 ^)^.

As for the time and cause of the absences according to the type of depression, there was also a statistically significant association for some subtypes. In South Korea, on the other hand, when the depression subtypes were compared, it was found that the differences between the types of severity of depressive symptoms (mild, moderate and moderately severe) and the mean days of leave of absence were significant. However, unlike the results of this research, there was no significant difference in the average number of days of absence of illness between groups with severe depressive symptoms^(^ 16 ^)^.

With regard to retirement due to disability associated with time, no studies were found with social security data that made this comparison. However, depression is recognized as a disabling disease, which can lead to permanent disability in those workers with persistent and severe depressive episodes and among those who have psychotic symptoms or greater cognitive impairment^(^ 24 ^)^.

Although no studies were found that made the relationship between retirements due to depression and long-term leave, it is confirmed that MBDs constitute the ones that most contribute to workers’ disability retirement and are among the groups of diseases that are configured among the most common in the Brazilian population^(^ 29 ^)^.

MBD, including depression, were mentioned among workers in a study carried out in the state of Mato Grosso do Sul, Brazil, in which, when logistic regression was performed, it was found that the age group variable was associated with psychiatric pathologies^(^ 30 ^)^.

In turn, in a survey conducted in the interior of the state of São Paulo, Brazil, with nursing professionals, it was found that depression is 3.27 times higher among those over 40 years old^(^ 31 ^)^.

Although the study on screen is not only about professionals in the aforementioned category, it is noteworthy that the result was significant for the age group. In this sense, when observing these data, it appears that absenteeism due to illness for longer periods, when related to age, plays an important role, since it demonstrates greater deterioration in the health status of this population.

In addition, the significant difference between time and subtypes of depression described reflects the adversities faced by workers, especially those who present psychotic symptoms, as these are associated with greater durability of symptoms, as well as a worse prognosis.

It is noteworthy, as a limitation, the use of secondary data, as well as the adoption of a cross-sectional design, since it makes it impossible to verify the previous health status of the worker, since the measures of interest are measured simultaneously, making it impossible to establish cause-effect relationships.

It also includes the fact that studies on this theme are still incipient, especially in Brazil, and that, when found, most of them address only health professionals.

It is considered, therefore, that the expansion of scientific knowledge about the absences motivated by depression can provide subsidies for the implementation of policies and actions directed at the workers’ mental health, as well as stimulating the realization of future investigations with a greater geographical range.

## Conclusion

The results of this study showed that the remote workers were characterized by being mostly female, with an average age of 47.2 years, living in the capital, Teresina, of urban origin, with income ranging from one to two wages minimums, with the majority departing only once. Social security sickness benefit was the type of benefit most granted, the time off was considered long, and the occurrence by type of depression in the studied population was more expressive for the mild depressive episode.

There was a statistically significant association for the variables age group, income and origin as well as for the mild depressive episode, severe depressive episode with psychotic symptoms, recurrent depressive disorder, current mild episode and recurrent depressive disorder and current serious episode with psychotic symptoms. As for the benefits, there was an association with accidental sickness benefit and retirement due to social security disability.
